# An Imaging Sensor-Aided Vision Navigation Approach that Uses a Geo-Referenced Image Database

**DOI:** 10.3390/s16020166

**Published:** 2016-01-28

**Authors:** Yan Li, Qingwu Hu, Meng Wu, Yang Gao

**Affiliations:** 1 School of Information Engineering, Chang’an University, Xi’an 710064, China; xaliyan72@163.com; 2 School of Remote Sensing and Information Engineering, Wuhan University, Wuhan 430079, China; 3 Xi’an Research Institute of Surveying and Mapping, Xi’an 710054, China; wumeng19nudt@163.com (M.W.); xagpsgy@163.com (Y.G.)

**Keywords:** vision navigation, imaging sensor, geo-referenced, image database, multiple sensor-integrated mobile mapping, image retrieval, image matching

## Abstract

In determining position and attitude, vision navigation via real-time image processing of data collected from imaging sensors is advanced without a high-performance global positioning system (GPS) and an inertial measurement unit (IMU). Vision navigation is widely used in indoor navigation, far space navigation, and multiple sensor-integrated mobile mapping. This paper proposes a novel vision navigation approach aided by imaging sensors and that uses a high-accuracy geo-referenced image database (GRID) for high-precision navigation of multiple sensor platforms in environments with poor GPS. First, the framework of GRID-aided vision navigation is developed with sequence images from land-based mobile mapping systems that integrate multiple sensors. Second, a highly efficient GRID storage management model is established based on the linear index of a road segment for fast image searches and retrieval. Third, a robust image matching algorithm is presented to search and match a real-time image with the GRID. Subsequently, the image matched with the real-time scene is considered to calculate the 3D navigation parameter of multiple sensor platforms. Experimental results show that the proposed approach retrieves images efficiently and has navigation accuracies of 1.2 m in a plane and 1.8 m in height under GPS loss in 5 min and within 1500 m.

## 1. Introduction

The objective of a moving platform is to provide accurate information for moving platforms, e.g., a land vehicle, an airplane, or aircraft in space, particularly when a global positioning system (GPS) or an inertial measurement unit (IMU) is not working effectively under poor environmental conditions [1,2]. Imaging sensors have been widely studied and applied to determine position and orientation via a technology called vision navigation. This approach may be effective for such applications because it can be utilized in a GPS/IMU-deprived environment (such as indoors, in urban canyons, and in far space) [3,4,5]. Accurate positioning and orientation in a downtown building forest district or a long-distance tunnel with GPS outage and longtime IMU drift is a significant challenge in 3D navigation of a multiple sensor-integrated platform even with high-performance GPS/IMU-integrated platforms, such as the land-based mobile mapping system (L-MMS) for 3D surveying and modeling. Imaging sensor-based vision navigation can avoid certain harsh environmental restrictions because this approach does not depend on any signal or radiant sources [1,6]. Nonetheless, this environmental condition becomes problematic when real-time and high-precision performance is required.

Imaging sensor-based vision navigation estimates position and orientation information according to the geometrical relation from overlapping sequence images, which can improve the reliability of a navigation platform. DeSouza and Kak [7] investigated the developments in vision-related fields for mobile robot navigation over the past 20 years. Vision navigation is classified into three different categories: map-based, map building-based, and mapless navigation [7,8,9]. Mapless vision navigation utilizes imaging sensors and does not consider any prior description of the environment. Sequence images from the imaging sensors are used for motion analysis to determine the relative position and orientation information of a moving platform. The recently developed simultaneous localization and mapping (SLAM) technique integrates imaging sensors to extract the 3D navigation information with which 3D environment data are used for a map building-based vision navigation application. SLAM is employed in self-driving cars, unmanned aerial vehicles, autonomous underwater vehicles, planetary rovers, new domestic robots, and even within the human body [10,11,12]. At present, imaging sensors are combined with other laser scanning sensors to obtain robust positions and orientations for SLAM-based applications. Mapless vision navigation is a relative navigation technology that supports moving platforms to understand surroundings and explore a local environment, such as in indoor navigation and self-driving cars. This type of navigation stores information from the current environment, as well as its own relative position in the environment.

Nonetheless, cases of absolute navigation with global 3D coordinates and orientation have been observed, such as outdoor navigation, the L-MMS for 3D surveying platforms, and space navigation [2,13,14]. These cases require global geo-referencing with highly accurate absolute 3D coordinates and may include GPS/IMU; however, harsh and poor environmental conditions limit the performance of GPS/IMU navigation. Thus, map-based vision navigation is implemented by providing a moving platform with a geo-referenced 3D model of the environment, such as a 3D map, landmark, and geo-referenced image database (GRID) [3,15,16]. Map-based vision navigation considers the map as a sensor to match the real-time imaging sensor and facilitate highly accurate absolute navigation in advanced driver assistance systems (ADAS). A collection of geo-referenced images serves as a map for real-time localization; this collection is normally treated as a 3D map because it contains not only a set of landmarks but also the corresponding 3D location information [17,18]. Thus, imaging sensor-based vision navigation has two application scenarios as a map-based navigation approach: one is to improve the reliability of navigation under harsh environmental conditions in which GPS and IMU are ineffective, such as in ADAS. The other is to improve navigation accuracy where the vision navigation result is considered to be new input for Kalman filtering with GPS/IMU, such as in L-MMS.

The key issues in image database-aided vision navigation are image searching and the matching methods with real-time images (RTIs) [19,20]. Results of recent research show that the process of matching two images of the same scene under different scales, illumination, and view angles has been developed successfully for years; nonetheless, several theoretical and technical problems must be addressed [21,22]. For example, two independent images share many similar features or pixels under certain circumstances, which may cause matching ambiguity given many candidate images. This condition is a serious problem for image database-based vision navigation. The speed of matching RTIs with the image database not only depends on feature computation but also on image search and retrieval. The organization model of the image database is combined with robust feature matching to realize matching with fast searching; these methods are the key techniques in GRID-based vision navigation.

Our paper presents a novel imaging sensor-aided vision navigation approach that uses the highly accurate GRID. First, the framework of GRID-aided vision navigation is established with sequence images derived from land-based, multiple sensor-integrated mobile mapping systems. Second, a highly efficient GRID storage management model is developed based on the linear index of a road segment for fast image search and retrieval. Third, a robust image matching algorithm is presented to search for and match RTIs with GRID; the matched image is then matched with the real-time scene to calculate the 3D navigation parameter of the multiple-sensor platform. Experimental results show that the proposed approach retrieves images highly efficiently and has navigation accuracies of 1.2 m in a plane and 1.8 m in height under GPS loss in 5 min and within 1500 m.

## 2. Methodology

### 2.1. Framework and Navigation Model of GRID-Aided Vision Navigation

**Figure 1:**
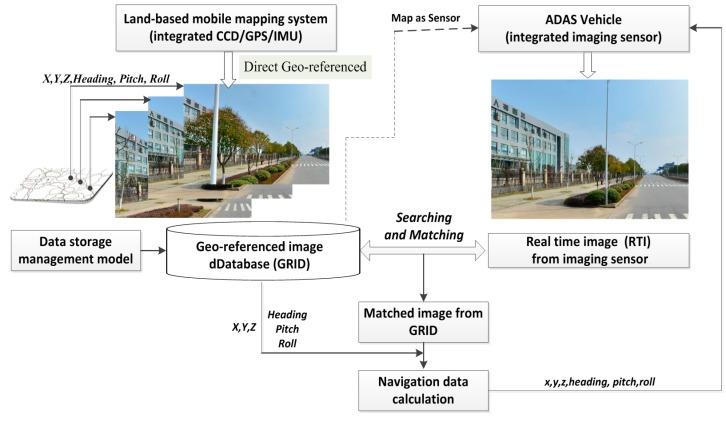
Framework of the proposed imaging sensor-aided vision navigation approach that uses a geo-referenced database.

The foundation of the proposed vision navigation approach is the comprehensive GRID with highly accurate position and orientation parameters that were derived from an L-MMS prior to vision navigation. All the images are captured and synchronized with the GPS/IMU according to image exposure time to calculate these parameters. The geo-referenced images are collected and are considered to be a highly accurate 3D survey of the environment. The imaging sensors of the moving platform capture the RTI of the current scene. Subsequently, this image is presented to match and search the GRID to obtain the exact geo-referenced image. Thus, the geo-referenced data are transferred to the RTI on the basis of the matched point with a strict photogrammetry geometry model to determine the 3D coordinates and the orientation parameter of the moving platform upon capturing the RTI. The framework of the proposed imaging sensor-aided vision navigation method with GRID is presented in Figure 1.

Three key issues are observed in the proposed vision navigation approach in relation to feasibility and accuracy; one is the performance of the image database. The geo-referenced image should be indexed well to reduce the time consumed in querying a candidate image set from the entire image database. Thus, we propose a data storage management model for GRID, as introduced in Section 2.2. The other issue involves the fast image searching and matching of the candidate image set with the RTI. The two processes should be completed quickly with high reliability, as described in Section 2.3. The third issue involves transferring the 3D navigation parameter from the geo-referenced image to the RTI precisely. We propose a vision navigation model from the image database to the moving platform, as explained in Section 2.4.

### 2.2. GRID Data Storage Management Model

#### 2.2.1. L-MMS for GRID Data Collection

**Figure 2:**
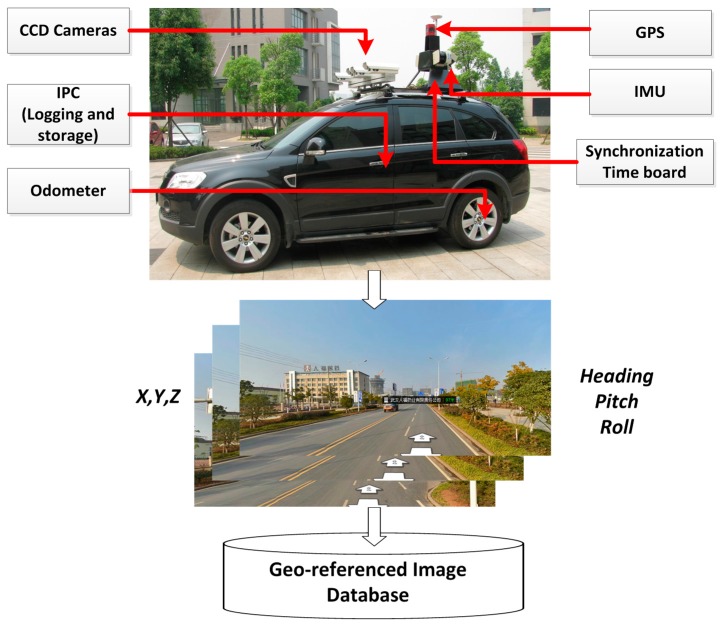
L-MMS for the data collection of geo-referenced database.

The GRID is compiled by multiple sensor-integrated, land-based mobile mapping systems, which were developed in the 1990s for rapid and efficient road surveying. Such systems include VISAT^TM^, GPS Vision^TM^, GI-EYE^TM^, LD2000^TM^, ON-SIGHT^TM^, Topcon IP S2, and Hi-Target iScan [2,23,24,25]. L-MMS integrates geodetic quality GPS, an inertial navigation system (INS), an odometer, a synchronization time board, and digital cameras that are mounted on a land vehicle (as presented in Figure 2). As a result, highly accurate GPS data can be logged along with INS orientation data and sequence images with high synchronized time in the process of running along defined routes at a speed of no more than 80 km/h.

Four or more cameras are installed in L-MMS to capture sequence images with a user-defined interval distance from 1 to 10 m. All the images are tagged with a time label by the synchronization time board, which adopts the GPS time as a time clock with an accuracy of less than 0.01 ms. The GPS/INS provides the orientation parameters for each sequence image based on the unique and unified GPS time. L-MMS is the most rapid, convenient, accurate, and economical surveying system for collecting and updating geo-information together with the GRID.

One L-MMS can obtain geo-referenced images for more than 200 km. Each image with a resolution of more than 2 MB in RGB mode has a data volume of approximately 5 MB. If the interval distance in image capturing is 5 m, then the number of images at 200 km will be 160,000 and the data volume will be 800,000 MB [2,26]. Determining the image number and the data volume are difficulties encountered in searching and matching RTI and GRID. The strategy and method of GRID design affects the efficiency of image searching and matching. To ensure the efficiency of the proposed vision navigation, we propose the indexing of a large number of images according to dynamic road section and the packing of images based on a large file storage model.

#### 2.2.2. Dynamic Section Indexing of GRID Based on Road Networks

At present, the quad-tree index is commonly used as a data organization and indexing method (such as the KIWI data format developed in Japan) [27,28] to navigate digital maps. The basic principle of this method is based on a quad-tree structure, and the geospace is divided into small grids. The map elements in the bottom grid to which the quad-tree leaf nodes correspond are packaged and stored according to location and size; furthermore, the map data are spatially separated into small blocks. The main advantage of the quad-tree index is its capability to quickly locate the current target area for a specified grid (block). The relevant target feature can also be extracted from an entire block. Nonetheless, obvious drawbacks are detected in GRID data indexing. GRID data are usually sampled along roads; therefore, data distribution in a quad-tree grid is uneven. As a result, the index data are redundant. In addition, all the images within the grid must be traversed and compared one by one in single-image retrieval (depending on the shooting location of the image), thereby lowering retrieval efficiency. Therefore, the GRID data indexing model in particular must be examined and designed.

GRID data indexing must satisfy two conditions. First, the traveling vehicle should be capable of quickly retrieving a recent image in a trend direction before reaching the current positioning point. Second, this approach should be capable of checking the image sequence in which the current positioning point of the region may be distributed during image matching and location retrieval. GRID data are linearly distributed along a road, unlike 2D GIS data with the geometrical features of point, line, and surface. Given this feature, a road-based dynamic segmentation (DS) index structure is designed in the linear referencing system to determine the spatial query speed of GRID and to ensure quick image matching.

The linear referencing system (LRS) constitutes a method of recording linearly distributed target position information. This system is widely used in the transport domain [29,30,31,32]. LRS can be expressed as (*R, M*), which are also known as linear coordinates that indicate the position information of linear characteristics in an relative offset *M*, *i.e.*, the distance to the starting point of the linear feature. *R* denotes the linear characteristic. For example, *(320, 274)* suggests that the distance from the starting point is *274* at road *320*. Essentially, the proposed DS index method does not alter the position of image distribution (shooting position). Moreover, the image space coordinate in the 2D reference system is not associated with a road in the linear referencing system to establish the GRID data index depending on the road network. The specific approach adopts DS technology according to the image distribution position and location section, applies the offset from the starting point of each road section representing the image position, and converts the image position from 2D coordinates *(X, Y)* to linear coordinates (*M*). Meanwhile, road split (non-physical division) is realized, and the image is hooked with the corresponding road section. A DS index (DS) tree is also built into the linear benchmark for each linear feature (road).

**Figure 3:**
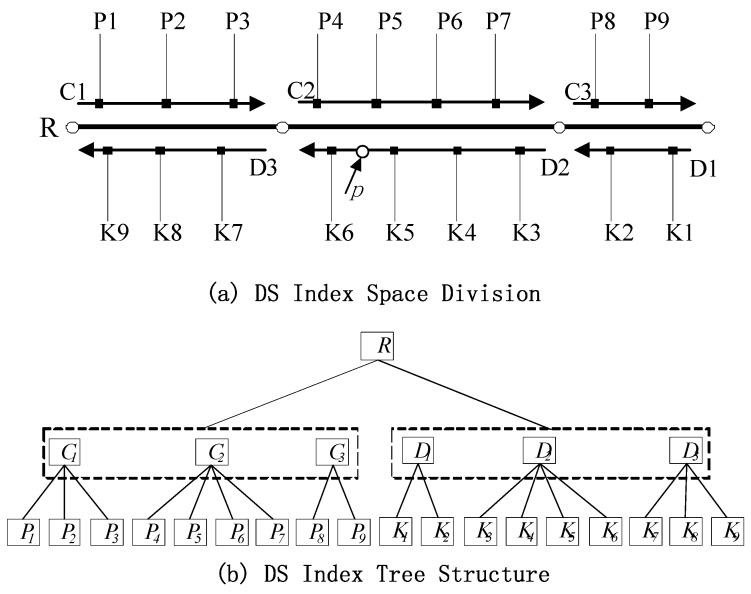
Dynamic segment indexing of GRID based on the road network LRS: (**a**) division of DS index space; (**b**) structure of the DS index tree.

The shape of the DS tree is similar to those of the *R* tree and quad-tree. However, the latter two indices are located in a 2D space, whereas the DS tree is indexed in a 1D linear space. In addition, the quad-tree and *R* tree represent the position of an object in an index space given the spatial coordinates of object (*X, Y*) or a minimum bounding rectangle, whereas the DS tree indicates the position of an object through offset M of the object. This tree is composed of root, intermediate, and leaf nodes. The 1D data set is divided recursively according to the nodes in each layer. In Figure 3, the two-direction road *R* includes two road-width-flow centerlines *C* and *D* that correspond to two road section sequences in opposite directions: *C1, C2, C3* and *D1, D2, D3*, respectively. These sequences comprise image sequences *P1, P2, …, P9* and *K1, K2, …, K9*. Figure 3a presents a schematic of the division of road R via DS index intervals. Figure 3b illustrates the corresponding DS index tree structure, where R is the root node. The structure of R can be expressed as R(*RID, M1, M2*). RID is the logical road index number. *M1* and *M2* are the beginning and ending offsets of the entire road. The starting offset of the road (*M1*) is generally set to 0. Intermediate node *C* or *D* includes section sequences along two directions of the centerline of road way flow, and the related structure can be expressed as S(*SID, M1, M2*). *SID* is the section index number, and *M1* and *M2* are the beginning and ending offsets of the sections along road flow, respectively. The leaf node stores information on each image in the GRID image sequence, and its structure is *I (IID, M)*. *IID* is the image index number, and M is the offset of the sections in which the image position is detected.

The data structure of the index tree node of GRID data is presented as follows:

  struct NODE
  {
  Linear_Interval linear;	//linear interval
  NODE*Parent;			      //a pointer to the parent node
  NODE**child;		//a pointer to the child node
  long*ObjID;		    //object ID within the linear interval
  }

		  

The quad-tree index in the 2D reference system must calculate the Euclidean distance between the location point and the image position individually for image retrieval. Meanwhile, the DS index in the linear referencing system only needs to compare the location point offset with the image position offset. The retrieval calculations are performed in a 1D space. Thus, the computation amount is reduced, and the retrieval efficiency can be improved.

#### 2.2.3. GRID Data Storage Based on Large Files

Many GRID images are collected by L-MMS. If 1 site includes four images and the distance between the two sites is 5 m, then the GRID data comprise more than 800,000 images given a city with 1000-km roads. If each image corresponds to a data file, then an excessive amount of files is produced, thus directly affecting data extraction efficiency. In addition, many small files induce frequent disk reading and writing, thereby resulting in straightforward disk damage. Therefore, the design of the data storage model for GRID must be optimized.

To effectively control the number of the generated GRID data files, a data storage model based on large files is proposed in this paper. The basic idea involves considering one road section as a unit to centrally store the image sequence in the section. This concept not only reduces the number of data files but also quickly determines the image sequence through a road section through the DS index and improves the efficiency of image retrieval.

The organization of GRID data based on large files is line with the following rules:

***Rule 1***: Images in one road section should be stored in one large data file as much as possible;

***Rule 2***: Cross-section images should be stored separately;

***Rule 3***: If the images in a section are so numerous that they constitute an excessively large image file (exceeding the set threshold), then this image file should be split and stored as multiple files.

**Figure 4:**
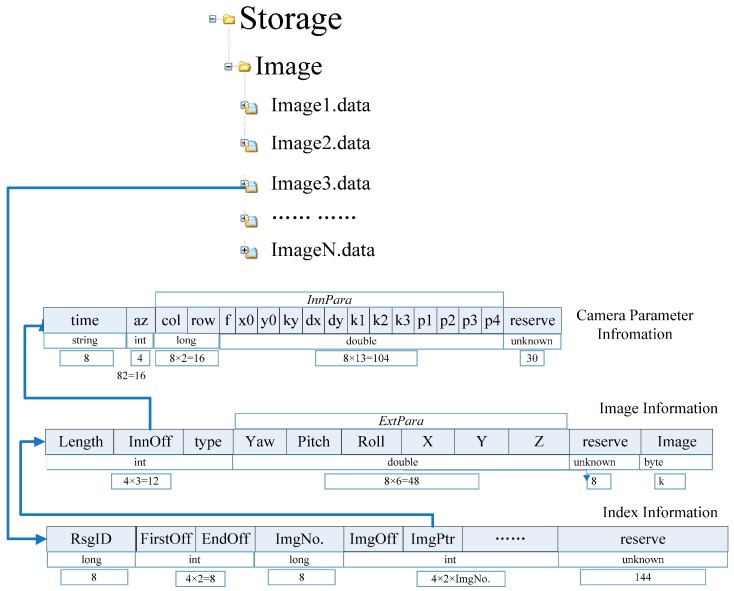
Large file-based data storage model for GRID.

The proposed large file-based data storage model for GRID is established through compliance with the aforementioned rules, as shown in Figure 4.

A large file for GRID can be divided into three parts: an image index table, a file body, and an image parameter file. The index table records the current file section ID (long type, eight bytes), first section offset of the image position in the file (int type, four bytes), final section offset of the image position (int type, four bytes), number of images (long type, eight bytes), section offset of each next image in turn (int type, four bytes), and stored address of the image in the file (int type, four bytes). The file body accounts for each image information record (44 bytes plus the space occupied by the image sequence). The image parameter file stores the interior and exterior orientations of the GRID. The size of each large file for this database should be controlled to below 200 MB for one road section. When the file size exceeds this recommendation, a new large file should be generated automatically. One record should not be stored in two separate files. If the current storage space is not enough to store an entire piece of data, then this information should be transferred to a new large file for storage. All large GRID files are labeled “Image” + n + “. data”.

The proposed GRID data storage based on large files can be organized in a database system or file directory, and this step is similar to image retrieval. In database storage, the generation of a large file can be regarded as the storage of sequence images related to a road section via a memory block. Meanwhile, the single file model can be treated as individual image storage.

### 2.3. Fast Image Searching and Matching

The RTI from a global GRID is impossible to search for and match efficiently or accurately. A navigation system typically has GPS and IMU sensors, which play the most important role in determining the position and orientation parameters of a moving platform. Imaging sensor-based vision navigation is adopted to enhance navigation reliability and accuracy for a moving platform in a harsh GPS or IMU-failed environment. Thus, the vision navigation module generates an initial navigation parameter from GPS/IMU. The initial position can restrict the range of GRID searching and matching, which can induce fast image searching and accurate matching. The proposed method to search and match the RTI from GRID includes three steps: searching for and locating large GRID files, extracting image features, and image matching.

#### 2.3.1. Searching for and Locating Large GRID Files

**Figure 5:**
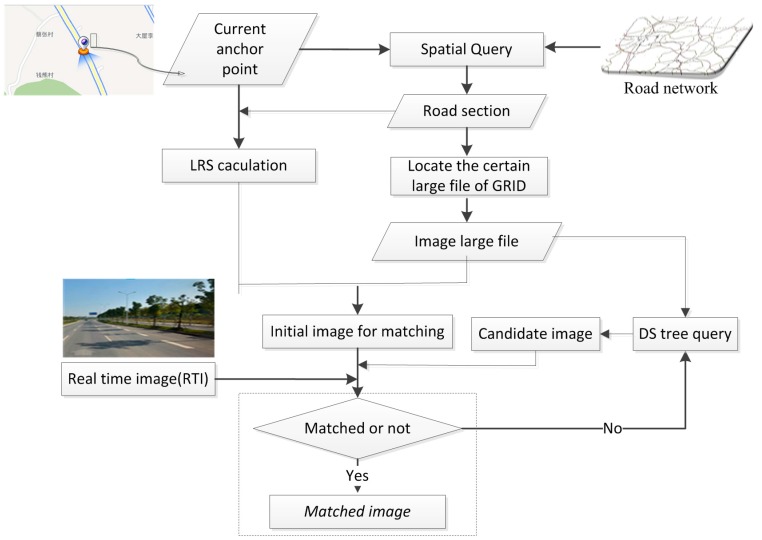
Fast search and large file location based on spatial query.

To obtain a fast and reliable search result from a high-volume GRID, a spatial query is implemented before image retrieval based on image understanding. Although the worst position accuracy of GPS/IMU is 100–500 m, it can restrict image searching and matching in a road section or a street block. This restriction can significantly reduce the workload of blind searching and matching over a wide range. Thus, a fast search that conducts a spatial query of the initial position can be performed through the proposed dynamic section indexing of GRID based on a road network. The road section of the initial position can be determined to locate a certain large GRID file for accurate matching. Figure 5 illustrates the proposed concept of fast searching based on spatial query to locate a large GRID file.

The current anchor point from GPS/IMU is considered as the spatial query input within the road network to determine the current road section. A large file can be located through the road section based on the proposed GRID data model. Meanwhile, the anchor point is converted into a 1D coordinate based on the LRS model to obtain the initial image from a large file as the candidate image for matching with the RTI. The candidate image is identified based on the DS tree; the process stops until the image is well matched with the RTI, as explained in the subsequent part.

#### 2.3.2. Extracting Image Features

RTI and GRID are the images captured in different environments of the same scene. Feature selection and extraction are the key issues in achieving highly reliable image matching. Lowe [33,34] proposed an efficient scale invariant feature transform (SIFT) algorithm that is widely used in image matching. The local features of SIFT include invariance in rotation, scaling, affine transformation, and a view angle, which is suitable for matching images captured at different times, distances, and viewing angles [33,34,35,36]. In the proposed approach, we adopt a 128-dimensional SIFT feature vector to describe the key feature point. The geometric distortion influence of scale and rotation can be reduced by this vector, and its normalization can eliminate the effect of illumination change. Thus, the SIFT vector of an image local feature can result in robust matching between RTI and GRID.

The SIFT 128-dimensional feature vector in GRID is time consuming to extract, particularly in real-time processing applications. To decrease the duration of matching between RTI and GRID, the SIFT feature vectors of each image in GRID are extracted in advance and linked to GRID.

#### 2.3.3. Image Matching

Image matching between RTI and GRID is implemented based on the SIFT feature. As this process runs during the image search, SIFT matching should be as fast as possible. The SIFT feature vectors of RTI can be extracted before matching because we have identified these vectors for each image in the GRID in advance. Thus, image matching is conducted only to calculate the similarity among the SIFT feature vectors of RTI and GRID. The SIFT matching algorithm usually adopts Euclidean distance as the similarity measurement of the key point. If the ratio of two key points with the closest Euclidean distance to the candidate key point for matching is less than a certain threshold (always taken as 0.9–1.0), then one of these two key points is considered a successful match. The number of matching points can be reduced by lowering the threshold ratio, thus stabilizing the SIFT matching process. In the proposed approach, the matching between RTI and GRID is simplified as the calculation of the Euclidean distance in the set of key points between RTI and GRID. A best-bin-first matching search algorithm with a 128-dimensional feature vector is presented as well, and all these strategies reduce the duration of searching and matching.

The correlation calculation based on Euclidean distance exhibits gross error. Handling mismatch is another important process during SIFT matching; normally, the SIFT matching algorithm follows a robust random sample consensus (RANSAC) [37,38] algorithm to address the gross error in SIFT matching.

**Figure 6:**
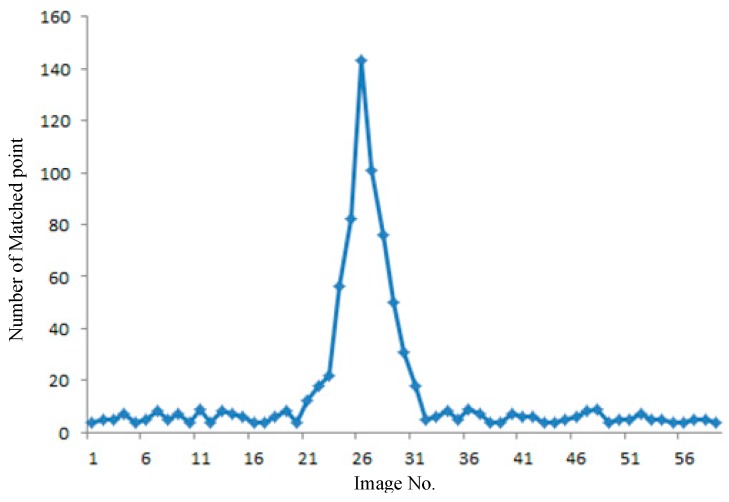
Fast search and large file location based on spatial query.

The image searching and matching processes intersect, as depicted in Figure 5. We can determine the match status according to the number of the matched key points between RTI and GRID. The number of matched points can be used to guide the search direction based on the LRS in a large file. Figure 6 presents an example of the number variance of the matched points. A total of 59 images from GRID are considered candidate images to match RTI.

The number of matched points increases when the GRID image is close to RTI. The entire matching process uses the same parameter of the SIFT algorithm and RANSAC. Thus, the matched image has the highest number of matching points. The number of matched points increases rapidly when the candidate image is in view of RTI; this step also helps determine the direction of image searching. The searching and matching time in all 59 images is 0.43 s; in real-time processing, these procedures stop after the image either matches well or fails. The searching and matching time is shorter than that of the SIFT matching of all the candidate images. The 59 GRID images cover a distance of approximately 300 m, which can be considered the largest error in the GPS/IMU position serving as the initial input for image interval retrieval.

### 2.4. 3D Navigation Calculation: From GRID to RTI

**Figure 7:**
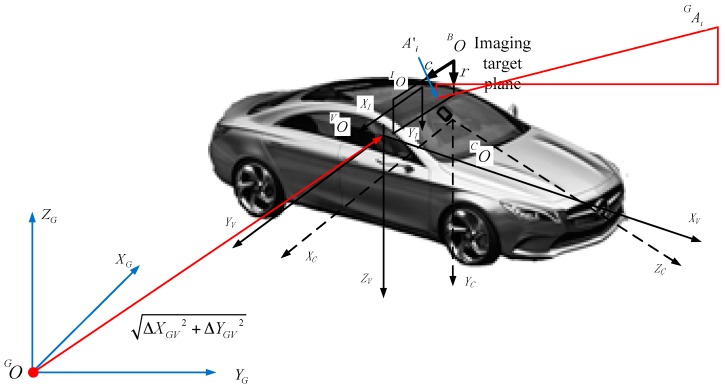
Definition of a coordinate system.

In the proposed imaging sensor-aided vision navigation system, a camera is fixed to the body of a moving vehicle, as described by a right-handed coordinate system. We define five coordinate systems to derive the navigation parameters from GRID to RTI, as shown in Figure 7.

${}^{G}P_{\left(X_{G},Y_{G},Z_{G}\right)}$ is the Cartesian coordinate of the geodetic coordinate system(GCS). $G_{O}$ is the coordinate origin and its coordinates in GCS are $P_{G}$, which is adopted by the GRID and the moving platform. ${}^{V}P_{\left(x_{V},y_{V},z_{V}\right)}$ is the body coordinate system(BCS) of a moving platform where the coordinate origin $V_{O}$ is defined as the intersection of two perpendicular lines of the cut planes through the center of gravity and the body; its coordinates in BCS are $P_{V}$. The $X_{V}$ axis is parallel to the longitudinal axis of the body, which is the forward direction of the vehicle. The $Y_{V}$ axis is perpendicular to the longitudinal axis of the vehicle body and parallel to the cutting plane, which is located to the right of the vehicle. The $Z_{V}$ axis is downward perpendicular to the cut plane. ${}^{C}P_{\left(x_{C},y_{C},z_{C}\right)}$ is the camera coordinate system(CCS), where coordinate origin $C_{O}$ is the projection center of the camera, and its coordinates in CCS are $P_{C}$. The $X_{C}$ axis is parallel to the direction of the camera scan lines and to the direction of the increasing scan pixels. The $Y_{C}$ axis is perpendicular to the direction of the camera scan lines and to the direction of the increasing scan lines. The $Z_{C}$ axis is perpendicular to the silicon target plane and to the gaze direction of camera imaging system. ${}^{I}P_{\left(x_{I},y_{I}\right)}$ is the image coordinate system (ICS) that defines the location coordinates of the optical imaging plane of the internal camera image, where coordinate origin $I_{O}$ is the focus on the image plane of the imaging system; its coordinates in ICS are $P_{I}$. The $X_{I}$ axis is parallel to the direction of the camera scan lines and to the direction of the increasing scan pixels. The $Y_{I}$ axis is perpendicular to the direction of the camera scan lines and to the direction of the increasing scan lines. ${}^{B}P_{\left(c,r\right)}$ is the pixel coordinate system(PCS) that defines the pixel coordinate, where coordinate origin $B_{O}$ is located in the top-left corner of the image; its coordinates in PCS are $P_{B}$. The c axis is parallel to the direction of the camera scan lines and to the direction of the increasing scan pixels. The r axis is perpendicular to the direction of the camera scan lines and to the direction of the increasing scan lines. Figure 7 indicates that ψ, θ, ϕ, ${}^{V}X_{C}$, ${}^{V}Y_{C}$, and ${}^{V}Z_{C}$ are considered to define the installation status of imaging cameras in the camera coordinate system.

The following paragraphs describe a few of the main transformations of the aforementioned coordinate system.

(*1*) *From the body coordinate system into the camera coordinate system*: the body coordinate system can be transformed into the pixel coordinate system. The transition from the former into the camera coordinate system includes translation and rotation, as indicated in Equation (1)

(1) $${}^{C}P_{\left(x_{C},y_{C},z_{C}\right)}={}_{V}^{C}R({}^{V}P_{\left(x_{V},y_{V},z_{V}\right)}-{}^{V}P_{C})$$

where ${}^{V}P_{C}$ is the coordinates of $C_{O}$ in the body coordinate system. ${}_{V}^{C}R$ is the rotation matrix, as per Equation (2)

(2) $${}_{V}^{C}R=R_{Y}(\psi )R_{X}(\theta )R_{Z}(\phi )$$

(*2*) *From the camera coordinate system into the image coordinate system*: this transformation is based on the principle of perspective projection, where the object distance is far greater than the focal length. Thus, the pinhole imaging model can replace the perspective projection model. The image coordinate can be calculated with Equation (3)

(3) $$\{\begin{array}{c} x_{I}=f\frac{x_{c}}{z_{c}}\\ y_{I}=f\frac{y_{c}}{z_{c}} \end{array}$$

(*3*) *From the image coordinate system into the pixel coordinate system*: the pixel coordinate system can be calculated from the image coordinate system through a 2D scale and translation, as indicated in Equation (4)

(4) $${}^{B}P=\left[\begin{array}{cc} N_{r} & 0\\ 0 & N_{c} \end{array}\right]\left[\begin{array}{c} x_{I}\\ y_{I} \end{array}\right]+{}^{B}P_{I}$$

where $N_{r}$ and $N_{c}$ are the numbers of rows and columns of width and length in the image coordinate system, respectively. ${}^{B}P_{I}={\left[{}^{B}C_{I},{}^{B}R_{I}\right]}^{T}$ is the offset of the main point position in the column and row directions.

In the transformation from Equations (1) to (4) explained above, ψ, θ, ϕ, ${}^{V}X_{C}$, ${}^{V}Y_{C}$, and ${}^{V}Z_{C}$ are usually called the outer orientation elements of an imaging camera. f, $N_{r}$, $N_{c}$, ${}^{B}C_{I}$, and ${}^{B}R_{I}$ are known as the inner orientation elements of such a camera.

*(4) From the body coordinate system into the* geodetic Cartesian coordinate system: the matched image is geo-referenced in the geodetic Cartesian coordinate system. All the matched key points have Cartesian coordinates ${}^{G}A_{i}\left(X_{GAi},Y_{GAi},Z_{GAi}\right)$ that are transferred to the corresponding points in RTI. Thus, we can convert ${}^{G}Ai$ to ${}^{V}Ai$ in the body coordinate system of the moving platform with Equation (5):

(5) $$\{\begin{array}{c} X_{GV}=X_{V}\cos\alpha +\Delta X_{G}\\ Y_{GV}=Y_{V}\cos\alpha +\Delta Y_{G}\\ Z_{GV}=-Z_{V} \end{array}$$

where $\Delta X_{GV}$ and $\Delta Y_{GV}$ are unknown quantities, and α is the heading of the moving platform. Then, we can convert ${}^{V}Ai$ to ${}^{C}Ai$ in the camera coordinate based on Equations (1) and (2). We can also transform ${}^{C}Ai$ into ${}^{I}Ai$ in the image coordinate system and ${}^{B}Ai$ in the pixel coordinate system on the basis of Equations (3) and (4). We finally complete the transformation of the key points ${}^{C}A_{i}\left(x_{CAi},y_{CAi},z_{CAi}\right)$ in the camera coordinate system to ${}^{B}A_{i}\left(x_{BAi},y_{BAi}\right)$ in the pixel coordinate system, as shown in Equation (6):

(6) $$\{\begin{array}{c} x_{BAi}=f\frac{x_{CAi}}{z_{CAi}}N_{r}{+}^{B}C_{I}\\ y_{BAi}=f\frac{y_{CAi}}{z_{C}}N_{c}{+}^{B}R_{I} \end{array}$$

The ${}^{B}{A^{\prime }}_{i}\left(x_{BAi},y_{BAi}\right)$ in the pixel coordinate system of RTI is determined through SIFT matching, and the corresponding ${}^{G}A_{i}\left(X_{GAi},Y_{GAi},Z_{GAi}\right)$ in the geodetic Cartesian coordinate system is also identified from the GRID. Both outer orientation elements (ψ, θ, ϕ, ${}^{V}X_{C}$, ${}^{V}Y_{C}$, and ${}^{V}Z_{C}$) and inner orientation elements (f, $N_{r}$, $N_{c}$, ${}^{B}C_{I}$, and ${}^{B}R_{I}$) can be effectively calibrated in a high-precision calibration site. Each pair of matched key points can generate two equations based on Equation (6), and we can build the equations with all the matched key points with unknown elements $\Delta X_{GV}$, $\Delta Y_{GV}$, and α. Thus, we can employ least squares’ adjustment to solve these unknown elements, which are the navigation parameters of the moving platform in the geodetic Cartesian coordinate system. To obtain a robust result, at least five key points should be used in the solving process.

## 3. Experimental Section and Discussion

The proposed imaging sensor-aided vision navigation approach has two key performance indicators that affect its actual application. One is the efficiency of the image searching and matching process, which is related to the GRID data organization model, and the other is navigation accuracy. We design two experiments to assess the proposed approach in terms of efficiency and accuracy. An L-MMS with six cameras is developed to collect GRID data; the GRID accuracies are 0.5 m in position and 0.3° in orientation. The GPS/IMU utilized in the L-MMS is SPAN^®^ IMU-FSAS™. The L-MMS is also used to verify the accuracy of the proposed vision navigation method based on GRID, whereas the high-precision GPS/IMU navigation data are considered the truth. One camera is simulated as the real-time imaging sensor.

### 3.1. GRID Efficiency Experiment

The GRID data organization method directly affects the efficiency of searching and matching between RTI and GRID. Thus, two experiments were designed to validate the proposed method. The computer is configured with a Pentium 4 2.0 GHz CPU, 1 GB memory, and a direct fiber 80 TB disk array with Windows XP Professional. We take three sets of GRID with data volumes that range from 1 GB and 100 GB to 1 TB by utilizing the proposed data organization model. This model represents the road, street (town), and county levels, as shown in Table 1.

**Table 1:** Experimental GRID data set.

Data Set	Data Volume (G)	Mileage (km)	Data Level
I	1.9	1.8	road
II	120.0	115.3	street (town)
III	1035.0	1027.7	county

#### 3.1.1. Comparison of the DS and Quad-Tree Indices for Image Spatial Query

The DS index converts the spatial query in 2D to a 1D space. Table 2 indicates the query response times after clicking the 2D navigation map as an image query input.

**Table 2:** Comparison of image spatial query times of the DS tree and the quad-tree.

Data Set	Index Model	Query Time (*s*)
I	Quad-tree DS tree	0.50 0.21
II	Quad-tree DS tree	0.52 0.22
III	Quad-tree DS tree	0.55 0.24

Image spatial query, which is based on the DS index, is faster than the quad-tree index. Furthermore, the proposed GRID data organization model does not differ significantly in terms of data volume because of the large file model. Each query is restricted to a specific large file.

#### 3.1.2. Comparison of Image Retrieval Efficiencies in Large and Small GRID File Storage Models

Image retrieval includes the image searching and image matching processes. Once the current searching image from GRID is matched to RTI, image searching ceases. We compare the image retrieval efficiency of the proposed large file model with that of the single-image file model. The average retrieval times of the three data sets are listed in Table 3.

**Table 3:** Comparison of image retrieval times in the large file model and the single-image file model.

Data Set	Index Model	Image Retrieval Time (*s*)
I	Single-image file Large file	1.25 0.78
II	Single-image file Large file	4.86 0.82
III	Single-image file Large file	18.24 0.86

The proposed large file model based on road sections is obviously advantageous over the single-image file model regardless of whether the files are stored in a database system or in a file directory. The image retrieval time of a large file model-based GRID is less than 1 s given three data sets. The retrieval time of the single-image file-based GRID increases with the data volume. The efficiency of the proposed GRID data organization model can meet the requirement of vision navigation based on this database.

### 3.2. Accuracy Analysis of GRID-Based Vision Navigation

**Figure 8:**
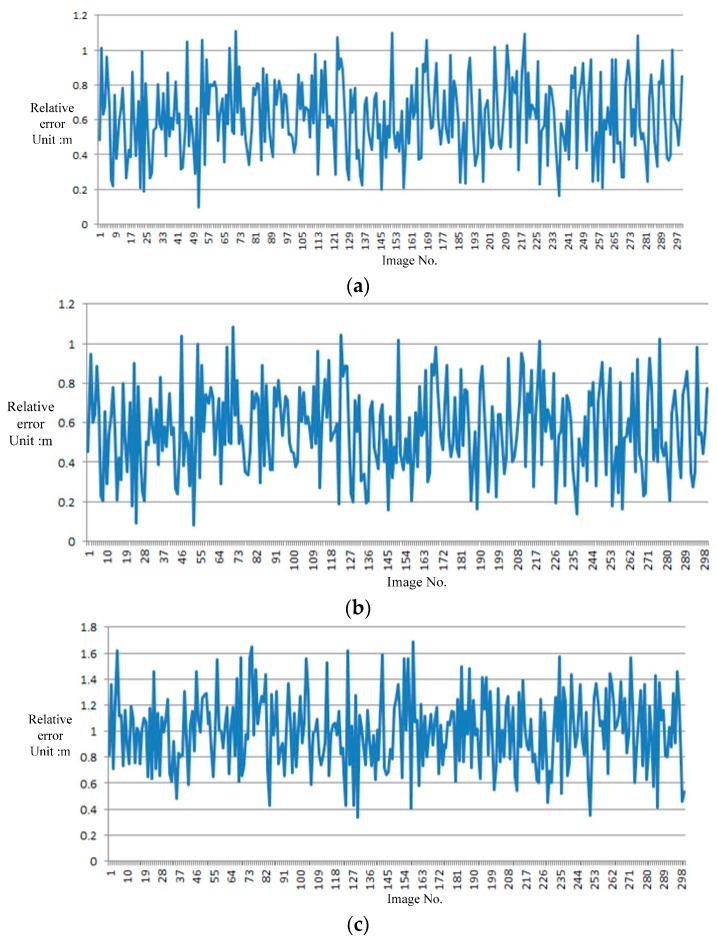
Accuracy of the proposed vision navigation approach: (**a**) *x* direction; (**b**) *y* direction; (**c**) *H*.

To assess the accuracy of the proposed vision navigation method, we use L-MMS to collect data on a single block twice at different times. One dataset is utilized to build the GRID as the proposed approach. Another dataset is employed to simulate RTI. A total of 300 images are regarded as RTIs for searching and matching with GRID. The image interval distance is set as 5 m, and the total time for 300 images is approximately 300 s within a distance of 1500 m. Then, the navigation parameters of the moving platform are calculated from the 300 images with the use of the proposed approach. The navigation results are compared with the tightly coupled integration of GPS/IMU via Inertial Explorer^®^ software. Figure 8 presents the absolute value of the relative error in *x*, *y*, and *H*.

The accuracy of the proposed imaging sensor-aided vision navigation method is less than 1.2 m in the x and y directions. *H* is less than 1.8 m. This accuracy is approximately double the accuracy of GRID because matching errors are detected in the key points between RTI and GRID.

### 3.3. Discussion

The use of a map as a sensor is a new ADAS trend. GRID is a map database that contains highly accurate position data and real environment information [39]; it can be used as a high-precision map database for ADAS. Although we have presented a basic framework of vision navigation with GRID, some challenges are still encountered for widely commercial applications in the navigation system. Nonetheless, the GRID data volume is extensive, as discussed in this paper; that of one county reaches the TB level. The current navigation database with a vector map always covers one county. Storing the high volume of GRID data on the navigation platform is a significant problem. The fast development of wireless Internet, such as 4G LTE and 5G LTE, is a way to realize online GRID services for vision navigation [40].

Although the GRID can provide real environment information for vision navigation, changes in road scenes are another challenge in imaging sensor-aided navigation. It is common that one road can change completely in a developing country. Such an altered scene complicates image retrieval and induces failure in certain cases. Rapid data collection and updating are important in GRID maintenance. The incremental update technology for a navigation map can also be used for this database. However, determining scene changes is particularly difficult in data collection and updating. Volunteered geographic information can harness tools to create, assemble, and disseminate geographic data provided voluntarily by individuals [41]; this information can serve as a crowdsourcing mode to determine scene changes for updating.

In fact, no error accumulates in the proposed technology. The reliability of localization after long periods of time depends on the image matching algorithms. Image matching is also a critical issue in GRID-based vision navigation [42]. Although we have introduced SIFT matching for RTI and GRID, challenges are still encountered with regard to matching speed and reliability, particularly given a wide image searching range. Such difficulties are experienced in vision navigation with low-cost GPS/IMU. Poor position inputs widen the range of image searching. Such a range worsens the reliability of image matching. The improved SIFT algorithms with multicore parallel computing [43,44] can be discussed further to improve the efficiency and reliability of this matching.

GRID-based vision navigation can be integrated with GPS/IMU, where the GRID can serve as a new sensor for federal Kalman filtering (FKF) in navigation calculation [45]. Our paper merely discusses vision navigation during GPS loss while one or more GPS satellites remain. Thus, we can further combine the result of vision navigation and of GPS/IMU based on FKF. The fusion navigation result can serve as vision navigation feedback to help narrow the searching range. The reliability and robustness of fusion navigation can also be enhanced with increased accuracy.

## 4. Conclusions

GRIDs are expanding worldwide with high resolution and accuracy, included Google Street View [46] and the Tencent Panoramic Image Service. The rapid development in global geo-referenced databases enables vision navigation with GRID. In this paper, we proposed a vision navigation approach based on this database to facilitate continuous and robust navigation for GPS and IMU sensors under harsh environmental conditions. The framework and navigation calculating model of GRID-aided vision navigation is established with sequence images from land-based multiple sensor-integrated mobile mapping systems. A highly efficient GRID storage management model is also developed based on the linear index of a road segment; large files are created to achieve fast image search and retrieval. This model can perform image searching and matching in 1 s regardless of data volume. A calculation method for 3D navigation parameters from GRID to RTI is also presented for imaging sensor-aided vision navigation with matched GRID images. The final result of vision navigation based on a GRID with 0.5 m accuracy indicates a 1.2 m accuracy in plane and 1.8 m accuracy in height under GPS loss in 5 min and within 1500 m. The proposed approach can be used to improve navigation reliability under harsh environmental conditions where GPS and IMU are ineffective, such as ADAS. When integrated with GPS/IMU, this method can also be employed to enhance navigation accuracy for L-MMS.
